# Fish oil supplements in patients with chronic kidney disease

**DOI:** 10.1093/ckj/sfag168

**Published:** 2026-05-27

**Authors:** Charles J Ferro, Mark R Thomas, Sophia Khattak, Dimitrios Chanouzas, Jonathan N Townend

**Affiliations:** Institute of Cardiovascular Sciences, University of Birmingham, Birmingham, UK; Department of Renal Medicine, Queen Elizabeth Hospital Birmingham, Edgbaston, Birmingham, UK; Institute of Cardiovascular Sciences, University of Birmingham, Birmingham, UK; Department of Cardiology, Queen Elizabeth Hospital Birmingham, Edgbaston, Birmingham, UK; Institute of Cardiovascular Sciences, University of Birmingham, Birmingham, UK; Department of Cardiology, Queen Elizabeth Hospital Birmingham, Edgbaston, Birmingham, UK; Department of Renal Medicine, Queen Elizabeth Hospital Birmingham, Edgbaston, Birmingham, UK; Institute of Immunology and Immunotherapy, University of Birmingham, Birmingham, UK; Institute of Cardiovascular Sciences, University of Birmingham, Birmingham, UK; Department of Cardiology, Queen Elizabeth Hospital Birmingham, Edgbaston, Birmingham, UK

**Keywords:** cardiovascular, CKD, fish oils, haemodialysis, inflammation, nutrition, omega-3 polyunsaturated fatty acids, oxidative stress

## Abstract

Cardiovascular disease (CVD) accounts for most of the increased morbidity and mortality associated with in chronic kidney disease (CKD). Cardiovascular risk emerges early in CKD, initially driven by atherosclerotic events but later dominated by heart failure and sudden cardiac death. This transition reflects progressive arteriosclerosis and the development of CKD-associated cardiomyopathy. Although patients receiving dialysis experience the highest cardiovascular risk, established atherosclerotic therapies such as lipid-lowering agents offer limited benefit at this stage. Recent therapeutic advances, including SGLT2 inhibitors, GLP1 receptor agonists and mineralocorticoid receptor antagonists, have improved outcomes in non-dialysis CKD, but the high cardiovascular mortality of dialysis patients remains largely unchanged, with multiple interventions having failed to demonstrate benefit. In this review, we outline the pleiotropic actions of omega-3 polyunsaturated fatty acids (PUFA) including anti-inflammatory, anti-oxidative, anti-thrombotic, plaque stabilising and membrane modulating effects. We discuss the PISCES trial that reported large reductions in cardiovascular events with high dose fish oil supplementation (1.6 g EPA + 0.8 g DHA daily) in 1228 haemodialysis patients followed up for 3.5 years. This treatment lowered events comprising the primary endpoint by 43% with similar reductions observed in cardiac death, myocardial infarction and stroke. Prior studies of fish oils in CKD have been small and inconclusive, underscoring the need for further large, randomised trials to confirm efficacy. If validated, omega-3 PUFA therapy could represent a long-needed advance for improving cardiovascular outcomes in haemodialysis patients.

## INTRODUCTION

Chronic kidney disease (CKD) is a major global health priority estimated to affect 13% of adults worldwide including close to 100 million Europeans, and projected to increase rapidly over the next decades [1–5]. The public health importance of CKD lies not just in progression to kidney failure requiring dialysis or transplantation but in its contribution to premature mortality, mainly from cardiovascular disease (CVD) [4–7].

**Figure 1:**
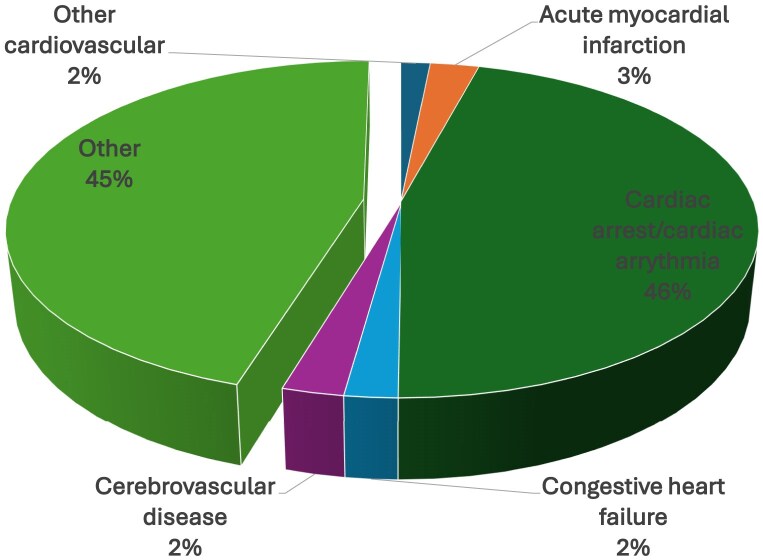
Causes of cardiovascular death in patients on haemodialysis as a proportion of known causes of death. Thirty-four percent of causes of death in haemodialysis patients are unknown. Data source, United States Renal Data System. 2025 USRDS Annual Data Report: Epidemiology of kidney disease in the United States. National Institutes of Health, National Institute of Diabetes and Digestive and Kidney Diseases, Bethesda, MD, 2025.

**Figure 2:**
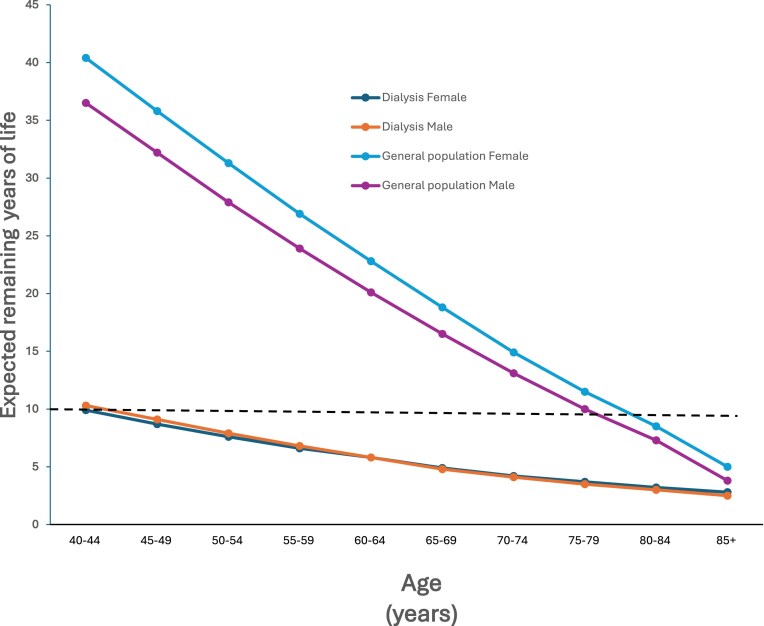
Expected remaining years of life in people with ESKD and the general population, by age and sex, in 2023. The dotted line highlights the fact that a dialysis patient aged 40–44 years has a lower life expectancy than a person in the general population aged 75–79 years. Data source, United States Renal Data System. 2025 USRDS Annual Data Report: Epidemiology of kidney disease in the United States. National Institutes of Health, National Institute of Diabetes and Digestive and Kidney Diseases, Bethesda, MD, 2025.ople with end-stage kidney d

Most of the increased morbidity and mortality associated with CKD is attributable to CVD [8, 9] with an increased risk beginning with both mildly reduced estimated glomerular filtration rate (eGFR) and very low levels of albuminuria (albumin: creatinine ratio >30 mg/g). This risk increases exponentially and independently with worsening eGFR and albuminuria with the highest risk being observed in individuals with severely reduced eGFR and high levels of albuminuria [6, 10, 11]. Not surprisingly, kidney failure, the most extreme category of CKD, is associated with the highest cardiovascular risk [12–14]. Patients on haemodialysis have an annual mortality of approximately 20% of which CVD accounts for around half (Fig. 1) [15, 16]. The increase in cardiovascular mortality compared to control populations is age dependent, varying from 2-fold in patients aged over 65, to over 100-fold in young adults [15]. Data from 2023 shows that a patient on dialysis aged 40–44 years has a lower life expectancy than a person aged 75–79 in the general population (Fig. 2) [16]. In the European Union, there are over 500 000 patients with kidney failure, and over 300 000 on dialysis, an increase of 150% in the last 25 years [17, 18].

**Figure 3:**
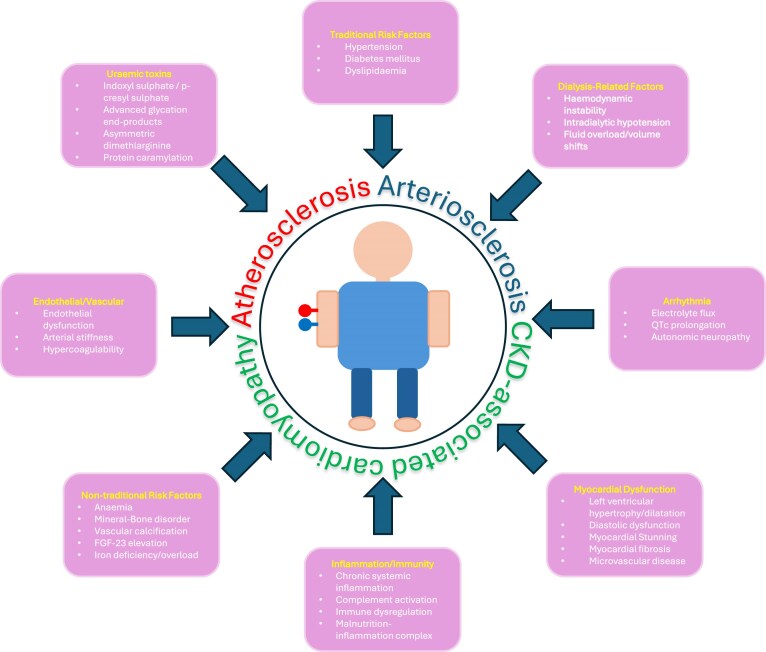
Postulated mechanisms of increased cardiovascular mortality in patients on haemodialysis, acting on the pathological processes of atherosclerosis, arteriosclerosis and CKD-associated cardiomyopathy. CKD, chronic kidney disease; FGF-23, fibroblast growth factor-23.

In the early stages of CKD, the risks of occlusive atheromatous disease are increased and account for most of the excess cardiovascular morbidity and mortality [12–14, 19, 20]. Arterial atheroma is an important modifiable pathophysiological process in the early stages of CKD as evidenced by multiple trials showing evidence of benefit from low density lipoprotein (LDL)-cholesterol reduction [20–23]. As CKD worsens there is a shift from atherosclerotic events to morbidity from heart failure and sudden cardiac death (SCD), with lipid-lowering therapies losing much of their efficacy [20, 22–25]. This change in the pathophysiology of CVD with worsening kidney function has been attributed to the development of both arteriosclerosis, characterised by conduit artery hypertrophy, stiffening and calcification [26–28], and CKD-associated cardiomyopathy: a triad of increased left ventricular mass, left ventricular diastolic and/or systolic dysfunction, and increasing interstitial myocardial fibrosis [8, 12–14, 20, 29–31]. These processes appear to be the result of multiple factors (Fig. 3). In dialysis patients, most cardiovascular deaths are attributed to SCD. Although few deaths are attributed to heart failure this may be an underestimate [16, 32]. Approximately 30–50% of patients on dialysis suffer from heart failure and even this figure is probably too low as the phenotype is usually ‘heart failure with preserved ejection fraction’ which can be difficult to detect in dialysis patients by imaging and the use of biomarkers [14, 33–35].

Over the last few years there have been very significant advances in the treatment of CKD. Agents such as SGLT2-inhibitors [36, 37], GLP-1 agonists [38], and finerenone [39, 40], a non-steroidal mineralocorticoid receptor antagonist, have been shown to significantly reduce both the progression of CKD and cardiovascular morbidity and mortality. In sharp contrast, novel treatments have largely failed to reduce the high mortality of dialysis patients. These include normalisation of haemoglobin concentrations [41], cinacalcet [42], non-calcium based phosphate binders [43], statins [23], and spironolactone [44–46]. Although haemodialfiltration with high-volume convection has been shown to reduce all-cause mortality [47], effects on cardiovascular mortality were suggested only by a meta-analysis incorporating data from four further trials [48] and these results were not without controversy [49].

### THE PISCES STUDY

**Figure 4:**
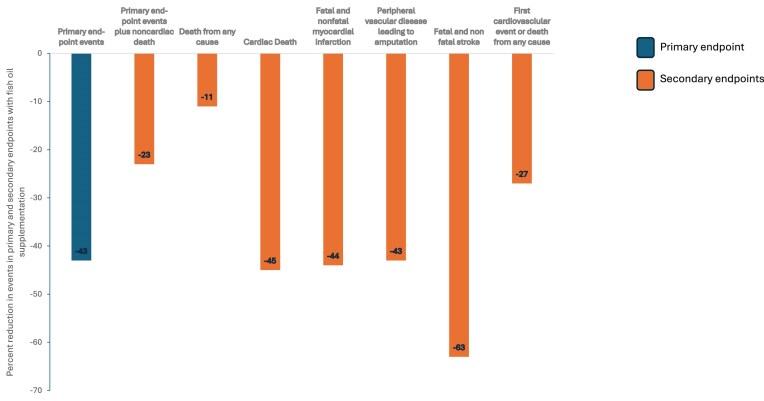
Point estimates for % reduction in events with fish oil supplementation in the Protection against Incidences of Serious Cardiovascular Events Study (PISCES). Data source: Lok CE, Farkouh M, Hemmelgarn BR, *et al*. Fish-oil supplementation and cardiovascular events in patients receiving hemodialysis. *N Engl J Med* 2026;**394**:128–137.

Against this background, the results of the Protection against Incidences of Serious Cardiovascular Events Study (PISCES study), published in November 2025, were both striking and surprising [50]. This prospective, randomised, double blind, placebo-controlled study assigned 1228 adults on haemodialysis to treatment with daily fish oil—using 1.6 g of eicosapentaenoic acid (EPA) and 0.8 g of docosahexaenoic acid (DHA) per day—or to corn-oil placebo. The primary endpoint was ‘serious cardiovascular events’ defined as a composite of cardiovascular death, myocardial infarction (MI), amputation due to peripheral vascular disease and stroke. Secondary end points were the individual components of the primary end point, non-cardiovascular death, a first cardiovascular event or death from any cause. Follow up was a median of 3.5 years. The results showed highly significant benefits from fish oil supplementation on CVD with effect sizes much larger than are seen in most modern trials (Fig. 4). Events comprising the primary endpoint were reduced by 43% and those of the extended primary end point by 33%. The effect of fish oil supplementation on many of the composite events could be described as astonishing with 45% reduction in cardiac death, 44% reduction in MI and a 63% reduction in stroke. Heart failure events were rare but further reduced by fish oil supplementation (1.6% vs. 2.3%). Unfortunately, data on SCD, as a key secondary end point were not given. All cause death rates were not significantly reduced (0.34 vs. 0.39 per 1000 patient days). The benefits were similar in patients with and without a history of CVD at recruitment.

### WHAT ARE FISH OILS?

The active agents in fish oils are polyunsaturated fatty acids (PUFAs) which exist as two major types: omega-3 (n-3 or ω-3) and omega-6 (n-6 or ω-6) [51]. PUFAs are distinct from saturated and monounsaturated fatty acids by containing two or more double bonds between carbon atoms in the fatty acid chain [51]. EPA, DHA, and linolenic acid are all omega-3 PUFAs with EPA and DHA being the most clinically studied.

Neither EPA nor DHA are considered essential nutrients but as human conversion of alpha-linolenic acid to EPA and DHA from is low, consumption of preformed EPA and DHA is recommended [52]. Both EPA and DHA are produced by marine microalgae which are consumed by fish. The content in fish is very variable depending on species, environment, and diet [51, 53–55]. Higher fat, cold water fishes such as salmon, herring, mackerel, sardines, and albacore tuna are rich in EPA and DHA. The cardiovascular benefits of fish oils were first postulated from epidemiological data from Japan and Greenland Inuits suggesting low rates of CVD [56–59]. This has been confirmed, by most but not all, subsequent prospective cohort studies [60–66].

The International Society for the Study of Fatty Acids and Lipids recommend at least 500 mg/day of EPA + DHA for the general population to maintain cardiovascular health [67]. This recommendation is consistent with UK Guidelines (450 mg/day as two portions of fish per week, one of which should be oily) [68], and American Heart Association (AHA) Guidelines for primary prevention [69]. The AHA Guidelines also recommend at least 1 g of EPA + DHA for secondary cardiovascular prevention [69]. The European Food Safety Authority recommends 250 mg/day of EPA + DHA as a primary preventive measure [55]. Despite these recommendations, European and UK intakes of EPA + DHA remain low [70, 71]. The Global Burden Study of Disease 2017 report found that a diet low in seafood EPA + DHA was the sixth leading dietary risk factor for mortality and disability-adjusted life years, especially through excess CVD [72]. In some countries this appeared to be more important than low fibre or high trans-fats diets [72]. Levels of omega-3 PUFAs in patients on haemodialysis are consistently lower than in the general population [73, 74].

Fish oil supplements are widely consumed but their content varies widely with some also containing saturated fatty acids and persistent pollutants including mercury which may have adverse effects [75–81]. In general, fish oil supplements have been studied at lower doses (typically 1 g/day), mainly to reduce cardiovascular events in high-risk patients, and at higher doses (2–4 g/day), mainly to lower triglycerides [20, 82–86]. It should not be overlooked that fish products contain other nutrients that might have cardiovascular and general health benefits such as calcium, magnesium, potassium, zinc, iron, and taurine [87]. Furthermore, the bioavailability of EPA and DHA is modified by the concomitant intake of fat and inorganic ions, as well as their chemical form, with lower availability of ethyl ester omega-3 PUFAs compared to triglyceride-bound fatty acids [88].

### WHAT DO FISH OILS DO?

**Figure 5:**
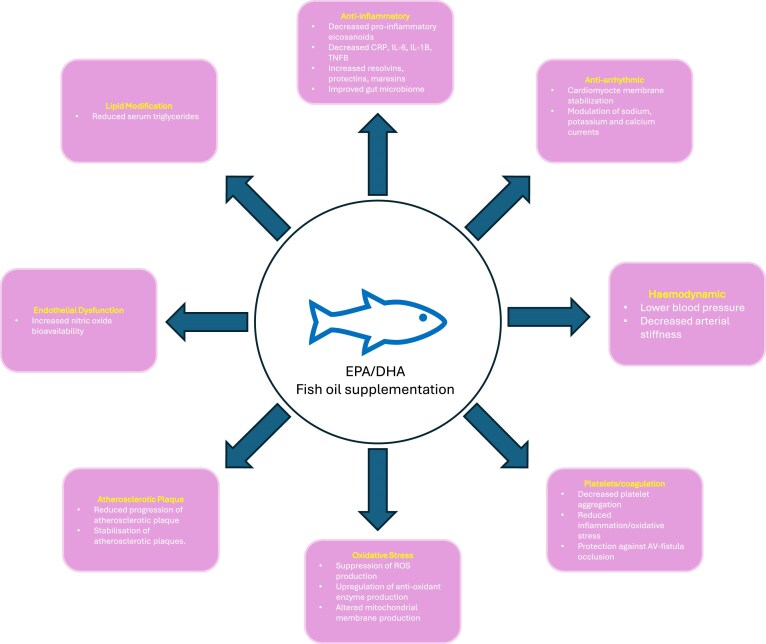
Actions of omega-3 polyunsaturated fatty acids with potential cardiovascular benefits for patients on haemodialysis. CRP, C-reactive protein; DHA, docosahexaenoic acid; EPA, eicosapentaenoic; IL-1B, interleukin-1β ; IL-6, interleukin-6; ROS, reactive oxygen species; TNFβ, tumour necrosis factor-β.

Omega-3 PUFAs alter the structure and function of cellular phospholipid membranes and have anti-inflammatory, anti-oxidative, membrane-stabilising, anti-thrombotic, and plaque stabilising effects (Fig. 5) [51]. In addition, high-dose omega-3 PUFAs lower circulating triglyceride concentrations [20, 86]. These inter-related effects are potentially responsible for the proposed cardioprotective actions of omega-3 PUFAs, though their relative importance is contentious.

### Inflammation

Chronic inflammation, as a maladaptive response, is associated with various diseases including CKD, CVD, obesity, and diabetes [89]. Inflammation is a key contributor to atherosclerotic plaque growth and rupture and a marker of risk in patients with atherosclerotic disease [90–93]. In CKD patients with atherosclerotic lesions, residual inflammation in the form of elevated CRP is a stronger risk factor than hyperlipidaemia in predicting recurrent cardiovascular events [94]. The inflammatory response to factors as diverse as infection and obesity is mediated by the production of cytokines and eicosanoids which are derived from the omega-6 fatty acid arachidonic acid or stimulated by its derivatives [95].

Both EPA and DHA lower circulating levels of interleukin-6, interleukin-1β, and tumour necrosis factor-α [54, 96–99]. They also reduce inflammation-related gene transcription by inhibiting the nuclear factor-kappa beta signalling pathway [54, 99]. Omega-3 PUFAs decrease circulating CRP concentrations in non-CKD patients with diabetes [100, 101]. DHA is also a precursor of specialized pro-resolving mediators, including resolvins, protectins, and maresins, which do not merely suppress inflammation, but actively initiate resolution of inflammation and tissue repair [54, 102–104]. EPA and DHA shift macrophages towards pro-resolving phenotypes [105]. By amplifying apoptotic cell clearance, this limits necrotic core expansion and stabilises atherosclerotic plaques [106]. In dialysis patients, fish oil supplementation has been shown consistently to reduce inflammatory markers [107–111].

The gut microbiome has been increasingly implicated in systemic inflammation and cardiovascular risk [89, 112]. Both EPA and DHA can modulate gut microbiota composition helping restore the Firmicutes to Bacteroidetes ratio, thereby increasing the production of the anti-inflammatory short chain fatty acid butyrate [113].

### Oxidative stress

Oxidative stress, resulting from an imbalance between reactive oxygen species (ROS) production and antioxidant defences, is closely linked with systemic inflammation and plays a significant role in the pathogenesis of atherosclerosis and metabolic disorders [114, 115]. In addition to exacerbating the inflammation associated with CKD, haemodialysis also increases oxidative stress due to mechanisms including loss of antioxidants during dialysis and activation of white cells [116].

Observational and interventional studies suggest that omega-3 PUFAs reduce oxidative stress [101, 117–119]. Although the mechanisms are not clear, several pathways have been proposed, including suppression of ROS production, upregulation of antioxidant enzyme expression (superoxide dismutase, catalase, and glutathione peroxidase) and alterations in mitochondrial membrane composition impacting on enhanced cellular defences against ROS [103, 120–125]. Fish oil supplementation has been shown to reduce markers of oxidative stress in haemodialysis patients [108, 110].

### Thrombosis

Both EPA and DHA dose-dependently inhibit platelet aggregation induced by arachidonic acid, adenosine diphosphate, and thrombin receptor activating peptide-6 [126–128]. In addition, omega-3 PUFAs may reduce thrombosis risk indirectly by reducing inflammation and oxidative stress, as well as improving endothelial function and altering the gut microbiome [128–132].

Although there have been historical concerns regarding the possibility of an increased bleeding risk associated with fish oil supplementation these appear to have been unfounded and several studies have shown a reduced bleeding risk [133, 134]. Patients with advanced CKD including those on dialysis paradoxically have both an increased risk of thrombosis and of major bleeding, attributed largely to platelet dysfunction [135–137]. So far there is no evidence that fish oil supplementation increases the risk of major bleeding in dialysis patients, indeed it may instead reduce this risk [50, 138].

### Anti-arrhythmia

Both EPA and DHA have been postulated to have pro- and anti-arrhythmic effects by virtue of their effects on the lipid raft environment, alteration of cell membrane structure and fluidity, modulation of sodium, potassium, and calcium currents, and regulation of gene transcription, cell proliferation, as well as anti-inflammatory effects [59]. Data from cell and animal studies support the hypothesis that omega-3 PUFAs reduce the risk of arrhythmia in patients during and after acute myocardial ischaemia where the mechanism of SCD is polymorphic ventricular tachycardia and ventricular fibrillation, not scar-based re-entry [139–148].

Many but not all observational studies have shown an inverse association between dietary omega-3 PUFA intake and SCD [58, 63, 66, 149–151]. The Gruppo Italiano per la Sperimentazione della Streptochinasi nell’Infarto Miocardico (GISSI)-Prevenzione trial, a large, open-label factorial RCT compared omega-3 PUFAs (850 mg EPA, 882 mg DHA) and vitamin E to placebo, in 11 324 patients with a recent MI. At 3.5 years, there was a 15% reduction in the primary end point (death, non-fatal MI, or nonfatal stroke) with EPA + DHA compared to controls, driven by a 45% reduction in SCD [152]. However, this reduction in SCD has not been replicated in more modern RCTs in which acute revascularisation is used and statins are widely prescribed [59, 153–161].

Both animal and experimental models have shown either a null or protective effect of omega-3 PUFAs on atrial fibrillation (AF) [162–173]. The relationship between omega-3 PUFA intake and incident AF in humans is however, far from clear [134, 174–186]. Most contemporary studies have demonstrated a dose-dependent positive association between AF and omega-3 PUFAs with sub-analyses suggesting high serum EPA levels, as a causal pathway for AF pathogenesis [54, 157, 159–162, 187–192]. A recent meta-analysis of seven contemporary randomised studies with 81 210 patients found a 25% increased risk of AF with PUFA supplementation compared with placebo over a weighted median 4.9 years of follow-up [148]. However, it should be noted that most trials have not adjusted for the competing risk of death, possibly inflating the risk of AF by informative censoring [148, 193].

Patients with CKD and especially those on dialysis have an elevated risk of AF [136, 194] but the effects of omega-3 PUFAs on this have not been extensively studied in these populations. The increasing evidence linking arrhythmogenic SCD in dialysis patients to brady-arrhythmias rather than VT/VF provide no support for any beneficial anti-arrhythmic effects of omega-3 PUFAs in this context [32, 195].

### Triglyceride lowering

High doses (2–4 g per day) of omega-3 PUFAs reduce triglyceride levels by up to 45% in a dose-dependent manner but have little to no effect on levels of LDL- or high density lipoprotein (HDL)-cholesterol in the general population or in patients with CKD or kidney failure [54, 84, 196–203]. The mechanisms of triglyceride lowering by omega-3 PUFAs are poorly understood and several pathways could be involved, including increased uptake into cellular phospholipids, reduced hepatic very low density lipoprotein (VLDL) triglyceride synthesis and/or secretion, enhanced triglyceride clearance from circulating VLDL particles, decreased hepatic lipogenesis and increased plasma lipoprotein lipase activity promoting degradation of apolipoprotein B [20, 86, 204–212]. In more severe hypertriglyceridemia, DHA, but not EPA, can raise LDL-cholesterol [51, 159, 199, 201, 210].

The characteristic pattern of dyslipidaemia in patients with advanced CKD and on dialysis consists of hypertriglyceridemia, low levels of HDL-cholesterol and variable but usually low levels of LDL-cholesterol and total cholesterol [20, 213, 214]. Several studies have reported that fish oil supplementation effectively lowers triglyceride levels in dialysis patients [215, 216].

### Atherosclerotic plaque

Omega-3 PUFAs are readily incorporated into atherosclerotic plaques given their lipophilic nature. Higher levels of EPA in plaques have been associated with decreased plaque inflammation and increased stability [91]. Supplementation with EPA (1.8 g/day) has been shown to increase fibrous cap thickness (reducing rupture risk) compared to baseline and to untreated controls in patients after an acute coronary syndrome [217]. The addition of EPA to intense statin therapy has also been shown to significantly increase plaque fibrous cap thickness and decrease plaque lipid arc and length compared with statin therapy alone [129, 218]. In patients awaiting carotid endarterectomy, those randomised to receiving omega-3 PUFAs had significantly lower mRNA levels of the proteinases responsible for fibrous cap thinning including MMP-7, MMP-9, MMP-12, and tissue inhibitor of MMP-12 compared with plaque from patients in the control group [91]. Treatment with EPA, either on its own or in combination with a statin, has been shown in multiple studies to reduce the progression of atherosclerosis in several patient groups compared with placebo or statin monotherapy [129, 219, 220]. To the best of our knowledge, no study has yet directly investigated the actions of fish oil supplementation on atheroma stability or progression in patients with CKD or on dialysis.

### Blood pressure

The effects of fish oils on blood pressure have been studied in several trials and reported in multiple meta-analyses [221]. Overall, fish oils administered in large doses (>3 g/day) lead to small, but meaningful reductions in blood pressure (∼1–6 mmHg systolic and 1–4 mmHg diastolic) [222–228]. The effects on blood pressure, as would be expected, are greater in patients with untreated hypertension, with higher doses and with greater duration of treatment [221]. The effects on blood pressure appear to be mediated by an increase in systemic arterial compliance and improvement in endothelial-dependent and endothelial-independent vasodilatation [221, 223, 229–235].

The actions of fish oil supplementation on blood pressure in patients with CKD or on dialysis have been much less studied and the reported effects are either small or non-existent [236].

### Biological aging

Accelerated biological aging is an increasingly recognised feature of CKD [237]. Indeed, CKD is associated with the greatest acceleration of biological aging among the 16 non-communicable disease studied so far [238, 239]. As biological aging is characterised by common cellular and molecular hallmarks representing shared cellular and molecular mechanisms, a rapidly expanding field of research explores ways to prevent and treat aging itself rather than the specific age-associated conditions [240]. Omega-3 PUFA supplementation is increasingly seen as a potential treatment for aging and aging related diseases [118] with accumulating evidence that they preserve telomere length [241–243] as well as preventing and reversing sarcopaenia [244].

## FISH OIL STUDIES FOR CARDIOVASCULAR RISK REDUCTION IN NON-CKD POPULATIONS

Evidence from RCTs has been inconsistent across eras and formulations (Table 1). Early secondary prevention studies, conducted largely before high intensity statins and comprehensive secondary prevention approaches, suggested benefit. In 1989 and 1999 respectively, the Diet and Reinfarction Trial (DART) and GISSI-Prevenzione studies showed that increased intake of oily fish or supplementation of omega-3 PUFAs after MI decreased major adverse cardiovascular events [245]. In contrast, multiple contemporary trials of omega-3 PUFAs have been neutral. From 2010 onwards, the Alpha Omega, the Outcome Reduction with an Intital Glargine INtervention (ORIGIN), the VITamin D and OmegA-3 TriaL (VITAL), and A Study of Cardiovascular Events in Diabetes (ASCEND) trials did not show any benefit in primary or secondary prevention populations [155, 188, 189, 246]. This led to a 2020 Cochrane review concluding that increasing omega-3 PUFA intake reduces triglycerides but only slightly reduces the risk of major adverse cardiovascular events [61]. Since then the Long-Term Outcomes Study to Assess Statin Residual Risk with Epanova in High Cardiovascular Risk Patients with Hypertriglyceridemia (STRENGTH), Omega-3 Fatty acids in Elderly with Myocardial Infarction (OMEMI), and Randomized Trial for Evaluation in Secondary Prevention Efficacy of Combination Therapy–Statin and Eicosapentaenoic Acid (RESPECT-EPA) trials have also produced neutral results [160, 161, 191].

**Table 1:** Summary of major randomised-controlled trials evaluating omega-fatty acid supplementation on cardiovascular events. Trials are listed in chronological order based on publication date.

Trial	Country/year/*N*	Patient population	Omega-3 type and formulation	Follow-up	Primary outcome definition	Adverse events reported
GISSI-Prevenzione [152]	Italy, 2002*N* = 11 323	Post MI patients (within 3 months)	EPA + DHA ethyl esters 1 capsule/day (EPA 290 mg + DHA 580 mg) Comparator: Vitamin E 300 mg/day OR Combination of EPA/DHA + Vitamin E OR Control	3.5 years	Combined death, non-fatal MI, non-fatal stroke. Reduction in composite end point and CV death.	GI upset, minor bleeding.
GISSI-HF [154]	Italy, 2008*N* = 6975	CHF NYHA II-IV	EPA + DHA ethyl esters 1 capsule/day (EPA 290 mg + DHA 580 mg) Comparator: placebo	Median 3.9 years	Co-primary end points: Time to death, Adjusted HR 0.91 95.5% CI 0.883–0.998; *P* = 0.041. Time to death or admission to hospital for CV reason. Adjusted HR 0.92 99% CI 0.849–0.999; *P* = 0.009	GI disturbance
JELIS [153]	Japan, 2007*N* = 18 645	Total cholesterol > 6.5 mmol/L	EPA ethyl ester 1800 mg/day (3 capsules) + statin Comparator: statin only Average dose Simvastatin 5 mg/day or pravastatin 10 mg/day Open label	Mean 4.6 years	Major coronary event: sudden cardiac death, fatal and non-fatal MI, unstable angina, PTCA/CABG. Significant reduction of events: 19% reduction *P* = 0.011.	GI disturbance, skin abnormalities, haemorrhages including cerebral and fundal bleeding, epistaxis, and subcutaneous bleeding.
ALPHA-OMEGA [155]	Netherlands, 2010*N* = 4837	Post MI patients aged 60–80 years on optimal therapy	4 trial margarines: i/EPA + DHA ii/ALA iii/EPA + DHA + ALA iv/placebo Additional intake of 226 mg EPA, 150 mg DHA and 1.9 g ALA or both in active groups.	Median 3.4 years	Composite of fatal and non-fatal CV events and cardiac interventions (PCI&CABG). HR for EPA-DHA 1.01, 95% CI 0.87–1.17; *P* = 0.93.	No difference between all 4 margarines
ORIGIN [246]	International, 2012*N* = 12 536	Dysglycaemia (impaired fasting glucose, impaired glucose tolerance, or early type 2 diabetes) plus ≥ 1 CV risk factor; >50 years old	EPA + DHA ethyl ester 1 g capsule (EPA 465 mg, DHA 365 mg) per day(Omacor/Lovaza) Comparator: olive oil.	Median 6.2 years	Death from cardiovascular cause. HR 0.98, 95% CI 0.87–1.10; *P* = 0.72	Abdominal discomfort
ASCEND [188]	UK, 2018 N = 15 480	Adults with type 1 or type 2 diabetes, no prior CVD at baseline;	EPA + DHA ethyl ester 1 g capsule (EPA 465 mg, DHA 365 mg) per day (Omacor/Lovaza) Comparator: olive oil.	Mean 7.4 years	First serious vascular event: non-fatal MI, non-fatal ischaemic stroke, TIA, vascular death. RR 0.97, 95% CI 0.87–1.08; P = 0.55.	GI symptoms. Excess serious bleeding (2.7% vs. 2.0) compared to placebo. Risk of AF not increased.
VITAL [189]	USA, 2019*N* = 25 871	Men > 50 years, women > 55 years. No prior CVD or cancer	EPA + DHA ethyl ester 1 g capsule (EPA 465 mg, DHA 365 mg) per day(Omacor/Lovaza) Comparator: placebo.2 × 2 factorial design with vitamin d 2000 iu/day	Median 5.3 years	Major CV event (MI, stroke, cv death) HR 0.92, 95% CI 0.80–1.02 *P* = 0.24.	No difference in adverse events compared with placebo
REDUCE-IT [159]	International, 2019*N* = 8179	Adults with established CVD or diabetes on statin therapy (fasting TG 1.52–2.53 mmol/L and LDL-cholesterol 1.06–2.59 mmol/L	EPA ethyl ester (Vascepa) 1 g capsules2 capsules twice daily (4 g/day) Mineral oil placebo.	Median 4.9 years	Composite of CV death, non-fatal MI, non-fatal stroke, coronary revascularisation, or unstable angina. HR 0.75, 95% CI 0.68–0.83; *P* < 0.001	Increased rate of AF in treatment group.
STRENGTH [160]	International, 2020*N* = 13 078	Statin treated patients at high CV risk with high TGs and low HDL-cholesterol	EPA + DHA carboxylic acids, 4 g once daily Comparator: corn oil placebo.	Median 3.5 years(Stopped early for futility)	Combined: CV death, MI, stroke, coronary revascularisation, unstable angina. No significant reduction in events (HR 0.99; 95% CI 0.90–1.09; *P* = 0.84)	High discontinuation rate due to GI intolerability. AF increased (2.2% vs. 1.3%; *P* = 0.001)
OMEMI [191]	Norway, 2021*N* = 1027	Patients aged 70–82 years 2–8 weeks post-MI	EPA + DHA ethyl ester 1.8 g (EPA 930 mg/DHA 660 mg)/day Comparator: corn oil placebo.	2 years.	Composite of nonfatal MI, unscheduled revascularisation, stroke, all-cause death, heart failure hospitalisation after 2 years. HR 1.08, 95% CI 0.82–1.41; *P* = 0.60	No significant difference with placebo. No increased risk of AF.
RESPECT-EPA [161]	Japan, 2024*N* = 2506	Patients 20–79 years, with stable CAD, on stable dose of statin, and EPA/AA ratio < 0.4	Highly purified EPA ethyl ester. 1800 mg/day in 2–3 divided doses. Open label	Median 5 years.	Composite of CV death, non-fatal MI, non-fatal ischaemic stroke, unstable angina requiring revascularisation, and coronary revascularisation. HR 0.79, 95% CI 0.62–1.00; *P* = 0.055	New onset AF higher in treatment group.
PISCES [50]	Canada and Australia, 2026*N* = 1228	Patients on maintenance haemodialysis	Fish oil (4 g of steam- deodorized, citrus-flavoured n-3 polyunsaturated fatty acids in four 1-g capsules containing a total of 1.6 g of EPA and 0.8 g of DHA) Comparator: citrus-flavoured corn-oil placebo	Median 3.5 years	Composite of CV death, non-fatal MI, non-fatal stroke, PVD leading to amputation. HR 0.57, 95% CI 0.47–0.70; *P* < 0.001	Lower risk of bleeding in fish oil group.

AA, arachidonic acid; AF, atrial fibrillation; ALA, alpha-linolenic acid; CAD, coronary artery disease; CHF, congestive heart failure; CI, confidence intervals; CVD, cardiovascular disease; CV, cardiovascular; DHA, docosahexaenoic acid; EPA, eicosapentaenoic acid; GI, gastro-intestinal; LDL, low-density lipoprotein cholesterol; MI, myocardial infarction; NYHA, New York Heart Association; HR, hazard ratio; TG, triglycerides; TIA, transient ischaemic attack.

There has been considerable speculation on the cause of these heterogenous clinical trial results, suggesting that baseline EPA/DHA levels, dose, and formulation may explain the discrepancies. The Japan EPA Lipid Intervention Study (JELIS) and the Reduction of Cardiovascular Events with Icosapent Ethyl–Intervention Trial (REDUCE-IT) studies both showed that EPA, without DHA reduces the risk of major adverse cardiovascular events [153, 159]. Icosapent ethyl is a highly purified, stable ethyl ester of EPA and does not contain DHA. This distinction is important, as EPA and DHA have different biological and clinical effects. EPA is incorporated into cell membranes where it alters membrane fluidity and lipid composition, influencing receptor signalling and inflammatory pathways. It also exerts antithrombotic effects without significantly impairing haemostasis [235, 247]. In contrast, DHA has been shown to increase LDL-cholesterol and may have less favourable effects on lipoprotein particle size [248]. In the Effect of Vascepa on Improving Coronary Atherosclerosis in People With High Triglycerides Taking Statin Therapy (EVAPORATE) study, icosapent ethyl significantly reduced low-attenuation plaque volume and total plaque burden on serial computed tomography [249].

The National Institute for Health and Care Excellence in the UK does not recommend routine use of fish oils after MI but has approved icosapent ethyl for cardiovascular risk reduction in patients with elevated triglycerides despite statin therapy [250]. The European Society of Cardiology lipid management guidelines also recommend icosapent ethyl as a Class IIa intervention in high-risk patients with persistently elevated triglycerides [251].

Together, these data suggest that heterogeneity in omega-3 PUFA trial outcomes may be, at least partly, explained by differences in formulation, dose, and biological activity [252]. Icosapent ethyl, as a purified EPA therapy, represents a distinct and evidence-based strategy for residual cardiovascular risk reduction in the general population with hypertriglyceridemia but requires further study in patients with CKD and kidney failure. This contrasts with the PISCES study where benefit was seen with a combination of EPA and DHA.

## PREVIOUS FISH OIL STUDIES IN CKD

Previous small studies of fish oils in patients with CKD provide limited support for the PISCES results. A 2020 systematic review and meta-analysis [253] of the effects of omega-3 PUFA intake in patients with CKD examined cardiovascular effects in eight trials (seven of patients on haemodialysis, one of kidney transplant recipients) consisting of 1104 participants with 40 events. The authors reported ‘low certainty evidence’ that omega-3 PUFA supplementation at a median dose of 2.7 g per day may decrease cardiovascular mortality (RR 0.44, 95% CI 0.23–0.86). Effects on major cardiovascular events were uncertain. Three studies (two in transplant recipients and 1 in haemodialysis patients) reported data on fatal or non-fatal MI. The relative risk point estimate was 0.39 but confidence intervals were very wide (0.15–1.05). A 2012 study aimed at assessing the effects of fish oil treatment (EPA + DHA, 4 g per day) on arteriovenous graft patency in 201 dialysis patients, reported that active treatment caused no significant effect on the primary outcome but was associated with better cardiovascular event free survival than placebo treated controls; the cardiovascular event rate per 1000 days was 0.39 in the fish oil group and 0.95 in the placebo group [254].

## THE PISCES STUDY: CRITIQUE AND IMPLICATIONS

Much effort has already been expended in attempting to explain the PISCES results, indeed they have been described as ‘too good to be true’ [255–257]. It is worth examining the trial in detail. The patient characteristics were representative of dialysis patients in their countries of recruitment with respect to age (mean 64 years, 24.9% over 75 years), ethnicity (40% white, 16% Asian, 13% Southeast Asian, 14% Black) and co-morbidity (diabetes 56%, hypertension 85%) but differ slightly from those in European countries in which the ethnic mix is more predominantly Caucasian. The prevalence of diabetes as the cause of kidney failure was 38.4% in PISCES which is probably reflective of most haemodialysis populations world-wide. Mean baseline LDL-cholesterol and triglyceride levels were low by most standards at 1.9 and 1.3 mmol/l, respectively. Only about one-third of patients had a history of CVD at recruitment which is perhaps lower than is seen in patients in most countries, but the results were maintained in patients with and without a previous cardiovascular event. About 55% of patients were on statin therapy, 50% were on beta blockers and 23% on anti-platelet or anti-coagulant drugs. At baseline, the percentage of incorporation of EPA + DHA was 4.7% which is probably representative of the 3%–5% seen in most ‘Western’ populations so it looks as if the patient group were not unusually low in fish oil consumption. Treatment discontinuation occurred in under 5% of patients with no differences in adherence to the trial regimen between the study groups. Fish oil treatment resulted in a significant rise in EPA and DHA incorporation values. Rates of total bleeding were 4.8% in the fish-oil group and 7.6% in the placebo arm negating suggestions of increased bleeding risk.

The dose of fish oils was high, and the regimen used was four times daily. Adherence to this multi-dose regimen is unlikely to be as complete outside a trial. Furthermore, other gastro-intestinal side effects including nausea and diarrhoea, as well as a fishy-after-taste with some preparations, may further limit tolerability and adherence outside of clinical trial conditions.

The trial population was not large by modern standards, and it is possible that some of the effects were due to random chance, but the results are consistent across different manifestations of CVD. Commentators have suggested the results are implausibly good and have called for independent confirmation [255–257]. The nature of the effects suggests a significant reduction in atherosclerotic events such as MI and stroke suggesting possible plaque stabilising and/or anti-thrombotic effects of fish oils. Importantly, the figures for SCD were not given so anti-arrhythmic actions are still speculative. If the current paradigm that states that most adverse cardiovascular events in patients with kidney failure are related to CKD-associated cardiomyopathy and not atherosclerosis is correct, it is likely that fish oils are also exerting actions upon the diseased myocardium that might include anti-inflammatory, anti-oxidant, and anti-fibrotic effects in addition to anti-arrhythmic effects.

The PISCES trial raises several questions. Firstly, can the results be independently confirmed in a further, preferably larger study? This has been strongly advocated for by other commentators [255–257]. Secondly, could similar results be obtained in other CKD groups such as patients with functioning kidney transplants, patients with low clearance who have not reached kidney failure and patients with even earlier categories of CKD? So far all of the studies done in these populations have been small and have examined surrogate outcomes. Thirdly, is the overall dose, ratio of EPA/DHA and formulation of omega-3 PUFAs used in the PISCES trial the optimum? It seems unlikely that this explains the apparently impressive results of PISCES as the dose and formulation differs little from other large recent trials (Table 1). Fourthly, are there other high-risk groups for whom fish oils could provide protection from CVD or does kidney failure represent a ‘sweet-spot’ for fish oil treatment? It is possible that many of the mechanisms that contribute to the very high cardiovascular risk of dialysis patients (Fig. 3) are mitigated by the multiple actions of omega-3 PUFAs (Fig. 5) and that the unexpected benefits observed in the PISCES trial are real but may be only applicable to dialysis patients. Finally, despite the impressive reductions in cardiac death and cardiovascular events, effects on all-cause mortality were not significant (HR 0.89, 95% CI 0.73–1.01), a reminder of the multiple risks contributing to the high mortality observed in haemodialysis patients.

If fish oils are to be adopted as standard of care for all dialysis patients there are the usual cost and feasibility problems to be overcome. There are also issues of sustainability. The seafood sector already faces challenges in providing a sustainable source of omega-3 PUFAs, including the negative effect of acidification due to climate change on fish stocks [258] and overfishing [259]. The growth of aquaculture to meet increasing demand has also led to environmental and welfare issues with poor management further contributing to habitat damage and climate change [260]. Reducing the amount of fish feed in farmed fish diets has led to a reduction in EPA + DHA content. In farmed Scottish salmon the amount of EPA + DHA decreased by 50% between 2006 and 2015 [53]. It is likely that alternative sources of omega-3 PUFA will be needed such as production from algal oils and other biotechnology derived sources to meet current and future needs sustainably [55, 261].

## CONCLUSIONS

The results of the PISCES study are both exciting and surprising. Further confirmation is required, preferably in at least one more, larger RCT, before omega-3 PUFA supplementation becomes standard of care in haemodialysis patients. Why this intervention appears so successful when so many others have failed so emphatically is not clear. Perhaps patients on haemodialysis have so many factors increasing their cardiovascular mortality that intervening in any single element makes little difference. The multiple actions of omega-3 PUFAs may provide at least part of the explanation. We are currently living through a ‘golden age’ in the treatment of non-dialysis CKD patients. Hopefully, the PISCES trial will stimulate the nephrological community to study not only fish oils in further trials but to examine other novel agents to give a better future to haemodialysis patients.

## Data Availability

No new data were generated or analysed in support of this research.
